# Non-Invasive Continuous Glucose Monitoring in Patients Without Diabetes: Use in Cardiovascular Prevention—A Systematic Review

**DOI:** 10.3390/s25010187

**Published:** 2025-01-01

**Authors:** Filip Wilczek, Jan Gerrit van der Stouwe, Gloria Petrasch, David Niederseer

**Affiliations:** 1 Institute of Emergency Medicine, Stadtspital Zürich Waid, 8037 Zurich, Switzerland; filip.wilczek@sanacare.ch; 2 GP Practice, Sanacare Gruppenpraxis Zürich Stadelhofen, Gottfried Keller-Strasse 7, 8001 Zurich, Switzerland; 3 Department of Cardiology and Cardiovascular Research Institute Basel, University Hospital Basel, University of Basel, 4031 Basel, Switzerland; jangerrit.vanderstouwe@usb.ch; 4 Hochgebirgsklinik, Medicine Campus Davos, Herman-Burchard-Strasse 1, 7270 Davos, Switzerland; gloria.petrasch@hgk.ch; 5 Department of Cardiology, Center of Translational and Experimental Cardiology (CTEC), University Heart Center Zurich, University Hospital Zurich, University of Zurich, 8006 Zurich, Switzerland; 6 Christine Kuehne Center for Allergy Research and Education (CK-CARE), Medicine Campus Davos, 7265 Davos, Switzerland

**Keywords:** continuous glucose monitoring, CGM, cardiovascular prevention, cardiovascular risk, healthy adults, non-diabetic, glycemic variability, postprandial glycemia, exercise, physical activity, diet

## Abstract

Continuous glucose monitoring (CGM) might provide immediate feedback regarding lifestyle choices such as diet and physical activity (PA). The impact of dietary habits and physical activity can be demonstrated in real time by providing continuous data on glucose levels and enhancing patient engagement and adherence to lifestyle modifications. Originally developed for diabetic patients, its use has recently been extended to a non-diabetic population to improve cardiovascular health. However, since data in this population are scarce, the effect on cardiovascular outcomes is unclear. CGM may offer potential benefits for cardiovascular prevention in healthy individuals without diabetes. The aim of this systematic review is to evaluate the use of CGM in healthy non-diabetic individuals, focusing on its potential to guide lifestyle interventions in the context of cardiovascular prevention, which may ultimately reduce cardiovascular risk.

## 1. Introduction

Cardiovascular disease (CVD) remains a leading cause of death globally (World health statistics 2024, [1]) and poses a large social and economic burden on healthcare systems, especially across the European Union (EU), with estimated costs of EUR 282 billion annually [1,2]. While diabetes is a well-known risk factor for CVD, non-diabetic individuals can also exhibit glucose fluctuations that contribute to cardiovascular risk. Higher and/or prolonged glycemic excursions are considered to have a negative effect on health due to an increase in inflammation, endothelial dysfunction, and oxidative stress [3,4,5]. Just like chronic hyperglycemia, augmented blood glucose fluctuations, even within the non-diabetic range, have been shown to influence cardiovascular risk. Emerging evidence suggests that acute glycemic variability can exacerbate oxidative stress, systemic inflammation, and endothelial dysfunction—pathophysiological processes that are central to the development and progression of atherosclerosis and other CVDs. These findings align with epidemiological studies that link higher glycemic variability to increased arterial stiffness, impaired coronary microcirculation, and elevated cardiovascular morbidity. By increasing reactive oxygen species production, they impair endothelial function. Endothelial dysfunction itself promotes the expression of adhesion molecules, so platelets and monocytes migrate into the intima, finally resulting in atherosclerotic plaques as a vascular complication [6]. In fact, increasing evidence indicates that significant variations in serum glucose levels may even be more deleterious than chronic sustained hyperglycemia regarding microvascular complications [7,8].

Continuous glucose monitoring (CGM) has become a valuable tool for measuring glucose fluctuations. The CGM systems consist of a small, wearable device that measures glucose levels in the interstitial fluid (ISF) just beneath the skin. The CGM sensor is inserted under the skin, usually on the abdomen or upper arm. The transmitter sends data to the receiver or a compatible smart device. The glucose concentration in the ISF of the subcutaneous tissue closely correlates with the glucose concentration in the blood. It is also pH stable and not susceptible to contamination. However, precision for very low and high glucose levels is still reduced [9,10]. Unlike traditional blood glucose monitors that provide a single reading at one point in time, CGM systems provide continuous data, giving more insight into trends and patterns of glucose levels. The nature of CGM also makes it useful for measuring acute changes in glycemia following interventions involving nutritional changes or physical activity [11].

Facing these aspects, CGM seems to be a viable tool for glycemic control, and yet, to the best of our knowledge, it has rarely been studied in non-diabetic healthy persons in the context of cardiovascular prevention. Thus, we will systematically review the data on the use of CGM in the high-risk setting of cardiovascular prevention in non-diabetic individuals.

## 2. Materials and Methods

This systematic review adheres to the Preferred Reporting Items for Systematic Reviews and Meta-Analyses (PRISMA) Statement.

### 2.1. Data Source, Search, and Study Selection

**Figure 1:**
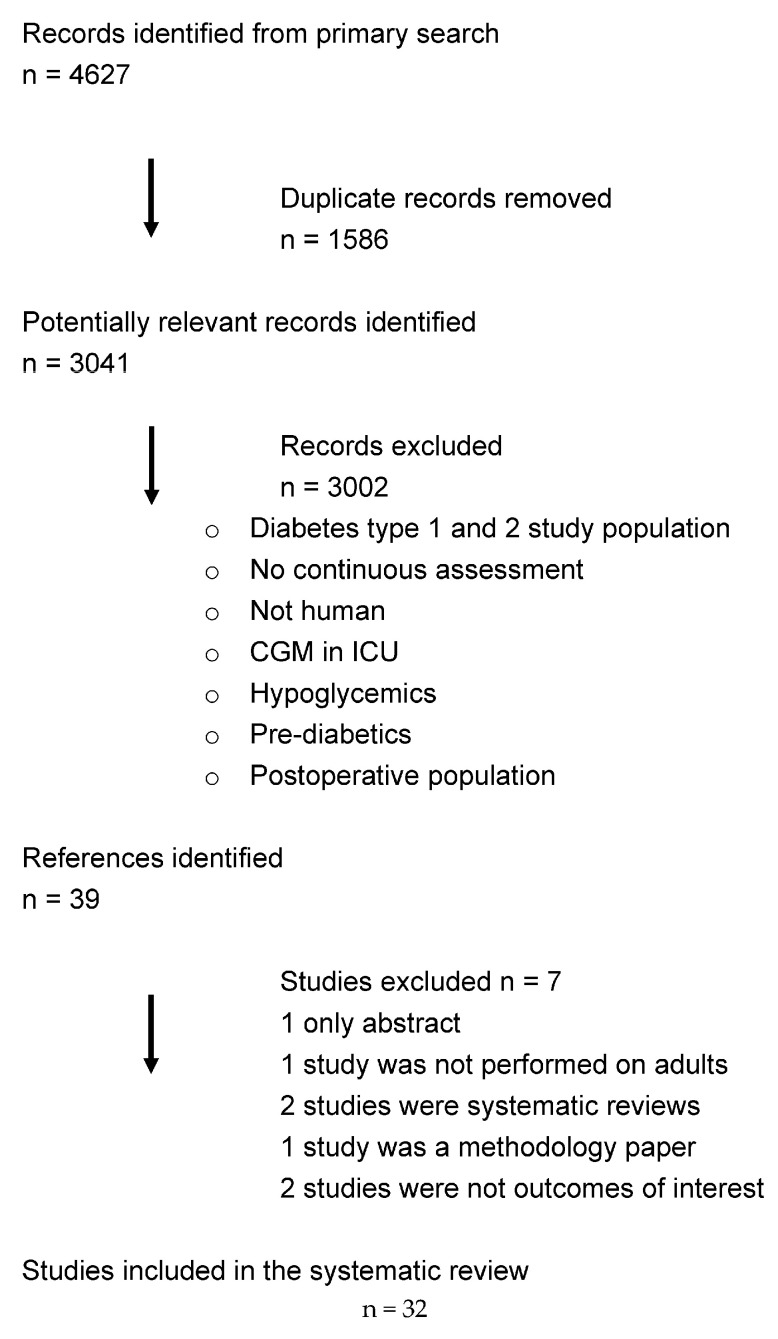
PRISMA diagram for study selection.

A comprehensive systematic search was conducted using the databases MEDLINE (via PubMed) and Embase for relevant articles, using a combination of the terms CGM and cardiovascular prevention (detailed description in the Supplementary Materials). The search was conducted on 13 March 2024. Details of the search are provided in Figure 1. Two reviewers independently performed screening of titles and abstracts and assessed the full texts for eligibility. Any discrepancies were discussed and resolved by a third reviewer.

Studies were included if they evaluated the impact of CGM on cardiovascular outcomes or related risk factors in non-diabetic, adult individuals. Studies pertaining to pregnant women or hypoglycemia, were excluded. We also excluded studies looking at postoperative cases and patients in the intensive care ward as well as reviews, meta-analyses, comments, and editorials.

### 2.2. Data Management and Extraction

After pooling all search results in a reference management software program (Zotero Version 7.0.6) [12] and removing duplicated results, two reviewers screened the titles and abstracts. A third member of the team resolved disagreements by discussion. Review of full text and final selection of studies was performed by two independent members of the study team. Disagreements were again resolved by discussion.

Then, the researchers carefully read the full text of the relevant studies based on the eligibility criteria (F.W. and D.N.). The authors (F.W. and D.N.) extracted essential information and entered it into the data extraction table. The extraction form contained items such as bibliographic information, type of study, participants and sample size, data source, (outcomes), and main results.

### 2.3. Data Synthesis

By aggregating the information extracted from each manuscript, we synthesized and organized the results from the included studies. We also explained the main results in narrative and tabular formats.

## 3. Results

A total of 3041 articles were identified during screening. After preliminary evaluation, 3009 articles were excluded. The remaining 39 full text publications were reviewed, and a further 7 studies were excluded. The remaining 32 studies were included in the present systematic review. The study selection process and reasons for exclusion are presented in Figure 1. Baseline study characteristics are presented in Table 1.

### 3.1. CGM, Glycemic Response on Nutritional Behavior

A total of eleven studies examined the association between nutritional behavior, such as mealtime, meal size, carbohydrate load, and the glycemic response. Nutrition is a vital part of cardiovascular prevention for all individuals, independent of baseline cardiovascular risk [13].

According to the results of three studies, a low glycemic index of a diet may influence the glycemic response. Buscemi et al. (2012) demonstrated that glycemic variability ameliorates with a hypocaloric diet with a low glycemic index (GI) and similar macronutrient and fiber content in nondiabetic obese persons. The glycemic index of the diet significantly influenced the 48-h glycemic variability measured as the coefficient of variability (CV%). The CV% decreased after the low GI diet and increased after the high GI diet [14]. Philippou et al. (2007) showed that patients with low GI diets had a significantly lower 24-h glucose profile compared to patients with a high GI diet in a 12-week randomized parallel study [15]. Also, Hafiz et al. (2022) showed that postprandial interstitial glucose levels were significantly higher after intake of instant mashed potatoes in comparison to all forms of chickpeas, which are known for having a very low GI [16]. Similarly, Li et al. (2018) examined the effects of daily consumption of quinoa-enriched bread on markers of CVD risk in healthy subjects, including plasma glucose. They found that daily consumption of quinoa in a short-term intervention appears to significantly lower glucose levels compared to wheat, which has a higher GI [17].

Furthermore, Kang et al. (2013) reported that the amount of carbohydrate in breakfast determines the postprandial glucose fluctuations within 180 min after meal ingestion. Postprandial glucose increased gradually with increased proportions of carbohydrate in breakfast in both impaired glucose regulation (IGR) subjects and normal glucose tolerance (NGT) subjects [18]. Also, Parr et al. (2018) concluded that for adults with prediabetes and prolonged sedentary periods, a low-energy first meal may be desirable for postprandial glucose and insulin regulation although 24-h glycemic control was not impaired by a high-energy first meal [19].

In contrast, Aston et al. (2009) observed no differences in glucose profiles between higher and lower GI interventions in participants, whose diet differed by a GI of 15 Units [20]. Likewise, Tey et al. (2017) showed that no significant differences were found in the mean 24-h glucose, iAUC, total AUC for glucose, and 24-h glycemic variability between beverages sweetened with non-nutritive (e.g., aspartame, monk fruit, or stevia) and nutritive (e.g., sucrose) sweeteners, although they differ strongly in their GI [21]. Kobayashi et al. (2013) reported that skipping breakfast increased the overall 24-h average of blood glucose [22]. Timmer et al. (2022) demonstrated that the glycemic response following consumption of a standardized carbohydrate-rich snack (a 65 g ginger-bread bar) differed over the course of the day. Mean glucose iAUC and mean glucose peak values were highest for snacks consumed between breakfast and lunch [23]. Jansen et al. (2022) showed that the adaptation from a low to a high carbohydrate diet may require many weeks, with implications for the accuracy of diabetes tests [24].

### 3.2. CGM, Glycemic Response on Physical Activity

Overall, ten studies examined the effects of physical activity (PA), with different types of exercise or exercise timing and/or duration on glycemic response. Current guidelines suggest regular physical activity reduces all-cause mortality, cardiovascular mortality, and morbidity in adults of all ages [13]. However, to what degree an improvement in daily glucose variation leads to improved outcomes in patients without diabetes is unknown.

Regarding the glycemic outcome, several studies showed a favorable effect of exercise. Zhang et al. (2023) demonstrated that both continuous (one 30-min walking bout) and accumulated (three 10-min walking bouts separated by 20-min rest) exercise reduced postprandial glucose (PPG) concentrations and decreased glucose fluctuations. Especially accumulated exercise maintained lower PPG concentrations for a longer time than continuous exercise in young obese adults [25]. Little et al. (2014) showed that high-intensity interval training (HIIT) may be an effective exercise strategy for improving glycemic control. A single session of HIIT in the morning improved postprandial glycemia control in overweight/obese adults for up to 24 h following exercise and was superior to continuous-moderate intensity (CMI) exercise [26]. Nakayama et al. (2022) reported that the glucose concentrations after meal intake (at 45 minutes, 60 minutes, and 75 minutes) and the iAUC were significantly lower in subjects with home-based high-intensity interval exercise (HIIE) and moderate-intensity continuous exercise (MICE) than in the control condition, without significant difference between both [27]. Smith et al. (2021) concluded that mean fasting glucose levels and daily glucose variation (CV%) reduced in frequent activity breaks from sitting (FABS, breaking sitting with three minutes of low-to-moderate-intensity physical activity every 30 min between 8 a.m. and 6 p.m.) [28].

Three studies demonstrated a reverse effect. Mikus et al. (2012) showed that during three days of reduced physical activity (<5000 steps/day), PPG significantly increased in healthy individuals [29]. Also, Trim et al. (2023) found that long-term physical inactivity (60 days of bed rest) increases glycemia (tAUC) [30], and Reynolds et al. (2023) showed that short-term (3 days) removal of exercise impairs glycemic control in older adults as well as young adults [31].

In contrast, Babir et al. (2023) examined the effect of an 11-min session of bodyweight exercise (BWE) on 24-h glycemic response, showing no differences between conditions for 24-h mean glucose, measures of glycemic variability, and the postprandial glycemic outcomes in comparison to a non-exercise sitting control period [32].

Two studies investigated the effect of exercise-meal timing on glycemic response. Solomon et al. (2020) showed that low- to moderate-intensity activity (30-min walking or bodyweight exercise) immediately after breakfast improved glucose control, while pre-breakfast activity or delayed post-breakfast activity did not affect mean glucose, CV, and AUC glucose [33]. Zhang et al. (2021) investigated the effect of post-meal exercise timing using an individualized approach on PPG in overweight or obese young men, demonstrating that walking initiated 20 min before postprandial glucose peak time (PPGP) lowered PPG concentrations [34].

### 3.3. CGM, Glucose and Cardiovascular Related Parameters

A total of eleven studies explored the association between glycemic variability (GV) and various physiological parameters, including physical constitution, biochemical and biophysical markers, sleep-related features, heart rate variability (HRV), and blood pressure variability (BPV). Salkind et al. (2014) found that normoglycemic and prediabetic morbidly obese individuals exhibit higher GV compared to normal-weight, non-diabetic individuals, suggesting a link/correlation between body composition/BMI and glucose fluctuations [35]. Buscemi et al. (2009) highlighted GV as a potential marker for assessing individual metabolic characteristics and the level of glycemic control. Furthermore, they identified interleukin-6 (IL-6), a proinflammatory cytokine, as an independent predictor for GV [36]. The ZOE PREDICT study by Bermingham et al. (2022) demonstrated that postprandial glucose responses and GV, as measured by continuous glucose monitoring (CGM), were less favorable in post-menopausal women compared to pre-menopausal women, indicating metabolic changes associated with estrogen decline during the menopausal transition [37]. Selvin et al. (2021) investigated CGM patterns in very old adults (range 77–91 years), observing that CGM parameters like mean CGM glucose and mean CV, including hypoglycemic events, differed substantially between the older participants, but mean CGM glucose strongly correlated with HbA1c, suggesting that CGM can provide supplementary information to HbA1c, especially in detecting hypoglycemia in both diabetic and non-diabetic populations. Additionally, they reported high feasibility and acceptability of CGM in very old adults [38]. Castaldo et al. (2011) evaluated the association between episodes of hypoglycemia and preclinical atherosclerosis in non-diabetic individuals with different degrees of glucose tolerance, showing that individuals with impaired glucose tolerance (IGT) spent significantly more time in hypoglycemia and exhibited higher carotid intima-media thickness (IMT) than glucose-tolerant individuals, indicating that hypoglycemia is a potential cardiovascular risk factor in individuals with IGT [39]. Also, Kuroda et al. (2015) demonstrated the impact of glucose fluctuations and hypoglycemia on coronary plaque properties. Both may contribute to lipid-rich plaque formation and fibrous cap thinning in coronary artery disease (CAD) patients [40]. Teshima et al. (2020) showed that chronic heart failure patients are at risk of hypoglycemia even without anti-diabetic therapy. These patients were exposed significantly longer to hypoglycemia compared to patients without chronic heart failure, underscoring the link between heart failure and glucose metabolism [41]. Rothberg et al. (2016) found a correlation between specific HRV patterns and blood glucose levels, but only in T2DM participants, indicating HRV as a potential determinant of glycemic control in these subjects [42]. Sezer et al. (2021) identified a significant correlation between daytime blood pressure values and GV in normotensive and normoglycemic healthy subjects, suggesting that both could serve as early markers of future adverse cardiovascular events [43].

Byun et al. (2020) demonstrated an association between the severity of obstructive sleep apnea (OSA) and changes in nocturnal blood glucose levels, showing that patients with moderate to severe OSA experience an increasing trend in glucose levels after sleep onset, in contrast to those without or with mild OSA, who experience a decreasing trend [44].

Finally, Liao et al. (2020) investigated the acceptability of CGM-incorporated physical activity intervention and changes in exercise motivation. They highlighted CGM as a feasible tool for exercise motivation in sedentary overweight and obese people, especially in the context of the transition from the pre-contemplation stage of behavioral change to the action stage. Additionally, they showed that acceptability ratings of CGM were comparable to those of an activity monitor wristband [45].

**Table 1:** Studies reporting results of non-invasive continuous glucose monitoring in patients without diabetes.

Author/Year	Type of Study; Country	Participants/Sample Size	Study Begin/End	Results
Aston et al., 2010 [20]	randomized, balanced, two-way crossover intervention; UK	12 overweight but otherwise healthy women, with a BMI of 25–30 kg/m^2^ and aged 18–65 years	May–December 2006	No differences were observed in glucose profiles between higher and lower GI interventions in the controlled or ad libitum setting.
Babir et al., 2023 [32]	randomized crossover trial; Canada	27 healthy, inactive participants, aged 18–35 years, inactive based on not meeting the aerobic physical activity targets in the Canadian 24 h Movement Guidelines for Adults	December 2021–May 2022	Mean 24-h glucose after BWE and CON was not different. No differences were found between conditions for measures of glycemic variability or the postprandial glucose responses after ingestion of a 75 g glucose drink or lunch, dinner, and breakfast meals.
Bermingham et al., 2022 [37]	randomized single arm, single blinded multicenter intervention trial; UK and US	1102 healthy adults, aged 18–65 years	June 2018–May 2019	Post-menopausal females had higher fasting blood measures compared with pre-menopausal females (*p* < 0.05 for all). Postprandial metabolic responses for glucose (2hiauc) and insulin (2hiauc) were higher (42% and 4% respectively) and CGM measures (glycemic variability and time in range) were unfavorable post- versus pre-menopause (*p* < 0.05 for all).
Buscemi et al., 2013 [14]	randomized controlled trial; Italy	40 participants, aged 18–60 years, BMI 25–49.9 kg/m^2^, with ≥2 diagnostic criteria of metabolic syndrome, except T1DM or T2DM	November 2010–February 2012	The glycemic index of the diet significantly influenced the FMD (*p* < 0.005). In particular, the change in FMD was 2.3 ± 2.6% following the LGI diet, and −0.9 ± 3.6% after the HGI diet (*p* < 0.005). The mean 48-h glycemia decreased significantly after dietary treatment (*p* < 0.05), but no significant effect of the glycemic index of the diet on results was observed. The glycemic index of the diet significantly influenced the 48-h glycemic variability measured as coefficient of variability (CV%; *p* < 0.001). The CV% decreased after the LGI diet (from 23.5 to 20.0%) and increased after the HGI diet (from 23.6 to 26.6%).
Buscemi et al., 2009 [36]	observational, three group study; Italy	28 overweight or obese subjects (11 males, 17 females), aged 30–60 years, BMI 25–35 kg/m^2^		The average CGM CV% increased from MS- group (21.1%) to the MS+ group (23.9%) and to the MS+/T2D group (27.4%), but it was not correlated with the CGM mean glycemia (r = 0.20; *p* = ns). Stepwise multiple correlation analysis showed that IL-6 predicted CGM CV% (R(2) = 0.35, beta = 0.13; *p* < 0.05) independently of BMI, waist circumference, adiponectin and insulin concentrations.
Byun et al., 2020 [44]	prospective, observational single center study; South Korea	23 patients, BMI < 35 kg/m^2^, no diabetes, no cardiac disease, no other sleep disorder		Those with moderate to severe OSA showed an increasing trend in blood glucose levels after sleep onset, whereas those without or with mild OSA showed a decreasing trend (F = 8.933, *p* < 0.001).
Castaldo et al., 2011 [39]	observational study; Italy	79 non-diabetic Caucasian subjects, aged 23–70 years, with no history of cv disease		IGT individuals had a worse cardiovascular risk profile, including higher IMT, and spent significantly more time in hypoglycemia than glucose-tolerant individuals.
Hafiz et al., 2022 [16]	randomized, crossover, controlled trial; UK	19 healthy adults aged 18–65 years, with fasting glucose < 5.6 mmol/L and BMI 18–29.9 kg/m^2^	15 August–20 December 2019	Postprandial glycemic responses were comparable between chickpea treatments, albeit significantly lower than the control *(p* < 0.001). All chickpea treatments elicited significantly lower C-peptide and GLP-1 responses compared to the control (*p* < 0.05), accompanied by enhanced subjective satiety responses (*p* < 0.05).
Jansen et al., 2022 [24]	randomized controlled trial; US	70 men and women aged 18–50 years, BMI ≥27 kg/m^2^ and no known cardiovascular disease or diabetes	May 2018–May 2020 (stopped 2019 due to COVID-19 pandemic)	Glucose metrics continued to decline after week 1 in the HC-Starch and HC-Sugar groups (*p* < 0.05), but not VLC. The number of participants with abnormal glucose tolerance by OGTT remained 10 (of 16) in VLC at start and end, but decreased from 17 to 9 (of 25) in both high-carbohydrate groups.
Kang et al., 2013 [18]	cross-sectional study; China	78 subjects with NGT and 55 IGR individuals 20–70 years of age, non-obese with BMI 18.5–24.9 kg/m^2^ without heart, lung, liver or kidney disease		The postprandial fluctuations of glucose increased gradually with increased proportions of carbohydrate in breakfast in both IGR and NGT subjects. Compared with the NGT subjects for the HC meal, the IGR subjects consuming the MC meal had greater PGS, range of glucose concentrations, SD, and PPGE (*p* < 0.05).
Kobayashi et al., 2013 [22]	randomized crossover trial with 2 experimental conditions; Japan	8 male young adults, free from pathological conditions, with no medication/supplements		Breakfast skipping did not affect 24-h energy expenditure, fat oxidation, or thermic effect of food, but increased the overall 24-h average blood glucose (83 ± 3 vs. 89 ± 2 mg/dL, *p* < 0.05).
Kuroda et al., 2015 [40]	prospective study; Japan	72 patients, referred for PCI for CAD, 20–80 years of age under adequate treatment for dyslipidemia, with LDL levels <120 mg/dL under statin administration or <100 mg/dL under other treatment for dyslipidemia	June 2012–April 2015	LI was stepwisely increased according to the tertile of MAGE (1958 ± 974 [tertile 1] vs. 2653 ± 1400 [tertile 2] vs. 4362 ± 1858 [tertile 3], *p* < 0.001), whereas FCT was the thinnest in the tertile 3 (157.3 ± 73.0 μm vs. 104.0 ± 64.1 μm vs. 83.1 ± 34.7 μm, *p* < 0.001, respectively). MAGE had the strongest effect on LI and FCT (standardized coefficient β = 0.527 and −0.392, respectively, both *p* < 0.001). Multiple logistic analysis identified MAGE as the only independent predictor of the presence of TCFA (odds ratio 1.034; *p* < 0.001).
Li et al., 2018 [17]	randomized controlled cross-over study; UK	37 healthy overweight (BMI > 25 kg/m^2^), non- smoking male volunteers, aged 36–70 years, with no known history of CVD or T2DM, and no medication	August 2016–February 2017	After 4 weeks of intervention, blood glucose and low-density lipoprotein (LDL) cholesterol were significantly lower than baseline in both groups, but there was no difference between quinoa and control. The cumulative area under the blood glucose curve for the last 4 days of the quinoa intervention tended to be lower than the first 4 days of washout (*p* = 0.054), and was significantly lower than the corresponding period of the wheat treatment (*p* = 0.039).
Liao et al., 2020 [45]	observational study; US	19 individuals (men and women), aged 18- 65 years, BMI ≥ 25 kg/m^2^, engaged in less than 150 minutes of moderate-intensity physical activity per week in the previous month		The summary acceptability scores for the self-monitoring period were 4.46 for CGM and 4.51 for Fitbit. Participants reported a significant decrease in the precontemplation stage and an increase in the action stage (*p* < 0.05).
Little et al., 2014 [26]	randomized, counter-balanced fashion; Canada	10 overweight or obese individuals (BMI >25 kg/m^2^, 8 females/ 2 males) who were inactive (<2 bouts of exercise lasting a minimum of 30 minutes per bout per week)		Exercise did not affect the PPG responses to lunch, but performing both HIIT and CMI in the morning significantly reduced the PPG incremental area under the curve (AUC) following dinner when compared with control (HIIT = 110 ± 35, CMI = 125 ± 34, control = 162 ± 46 mmol/L × 2 h, *p* < 0.05). The PPG AUC (HIIT = 125 ± 53, CMI = 186 ± 55, control =194 ± 96 mmol/L × 2 h) and the PPG spike (HIIT = Δ2.1 ± 0.9, CMI = Δ3.0 ± 0.9, control = Δ3.0 ± 1.5 mmol/L) following breakfast on the following day were significantly lower following HIIT compared with both CMI and control (*p* < 0.05). Absolute AUC and absolute glucose spikes were not different between HIIT, CMI, or control for any meal (*p* > 0.05 for all).
Mikus et al., 2012 [29]	observational cross sectional study; US	12 healthy volunteers (8 men, 4 women); 20–35 years of age, generally healthy (determined by detailed medical history questionnaire), recreationally active (≥ 10.000 pedometer steps per day)		During 3 d of reduced physical activity (12,956 ± 769 to 4319 ± 256 steps per day), PPG increased at 30 and 60 min after a meal (6.31 ± 0.19 to 6.68 ± 0.23 mmol/L and 5.75 ± 0.16 to 6.26 ± 0.28 mmol/L, *p* < 0.05 relative to corresponding active time point), and ΔPPG increased by 42%, 97%, and 33% at 30, 60, and 90 min after a meal, respectively (*p* < 0.05). Insulin and C-peptide responses to the OGTT increased after 3 d of reduced activity (*p* < 0.05), and the glucose response to the OGTT did not change significantly.
Nakayama et al., 2022 [27]	randomized crossover trial; Japan	12 healthy young adult males, with postprandial hyperglycemia defined as blood glucose level >140 mg/dL at 30 or 45 min after a meal		The glucose concentrations after the meal were significantly lower in the home-based HIIE and MICE conditions than in the control condition (*p* < 0.001). There were no significant differences in the glucose concentrations between the home-based HIIE and MICE conditions.
Parr et al., 2018 [19]	randomized, crossover study; Australia	13 men and women aged 40–70 years with overweight/obesity, sedentary and lifestyles with IFG and/or IGT	February–September 2016	Total glucose area under the curve (AUC; +5.7 mmol/L/h, *p* = 0.019) and mean plasma glucose concentrations (+0.5 mmol/L, *p* = 0.014) were greater after HE-BF compared to LE-BF. In the HE-BF condition, compared to LE-BF, there was a greater incremental area under the curve (iAUC) for plasma glucose post-breakfast (+44 ± 59%, *p* = 0.007), but lower iAUC post-lunch (–55 ± 36%, *p* < 0.001). Total insulin AUC was greater (+480 mIU/mL/h, *p* < 0.01) after HE-BF compared to LE-BF. Twenty-four-hour (24 h) CGM revealed no differences in mean glucose and total AUC between conditions.
Philippou et al., 2008 [15]	randomized parallel group trial; UK	13 subjects, aged 35–65 years and one heart disease risk factor (BMI 27–35 kg/m^2^, waist ≥ 88 cm females or ≥94 cm males, cholesterol: HDL ratio ≥ 5.0 mmol/L, BP systolic > 130 mmHg or diastolic > 85 mmHg)		A significantly different dietary GI was achieved in the low GI (median: 51.3 (IQR: 51.0–52.0) compared to the high GI (59.3 (59.2–64.0) (*p* = 0.032) group. By week 12, the low GI group also had a significantly lower 24-h area under the curve (AUC) (7556 (7315–8434) vs. 8841 (8424–8846) mmol-h/L (*p* = 0.045) and overnight AUC (2429 (2423–2714) vs. 3000 (2805–3072) mmol-h/L (*p* = 0.006) glucose as measured by CGMS.
Reynolds et al., 2023 [31]	randomized, crossover, exploratory study; US	20 participants (11 old, 9 younger), aged >55 years or between 18–40 years completing at least 30 min of moderate to vigorous exercise on 3 or more days per week during the past 3 months		Significant main effects of age (*p* = 0.002) and time (*p* < 0.001) existed for 1-h PPIG, but no effect of phase or interactions was found (*p* > 0.05). Significant main effects (*p* < 0.05) of age (old: 114 ± 1 mg/dL, young: 106 ± 1 mg/dL), phase (NOEX: 112 ± 1 mg/dL, EX: 108 ± 1 mg/dL), and time (0 min: 100 ± 2, 30 min: 118 ± 2, 60 min: 116 ± 2, 90 min: 111 ± 2, 120 min: 108 ± 2 mg/dL) in 2-h PPIG were detected, but no interaction was found (*p* > 0.05). Only significant main effects of phase (NOEX: 14 ± 1 and EX: 12 ± 1, *p* > 0.05) were found for 24-h blood glucose standard deviation.
Rothberg et al., 2016 [42]	cross-sectional, observation study; Australia	32 subjects with DM, and 31 subjects without diabetes (without history of cv events)		Low-frequency (LF) power, high-frequency (HF) power, and total power (TP) of HRV were negatively associated with BGL in participants with DM. The ratio of LF to HF was positively correlated with BGL.
Salkind et al., 2014 [35]	uncontrolled, observational study; US	36 morbidly obese (BMI ≥ 40 kg/m^2^) applicants of the TV show The Biggest Loser		Morbidly obese prediabetic subjects (n = 15) had GV metrics indistinguishable from those morbidly obese subjects who were normoglycemic. Normoglycemic and prediabetic morbidly obese individuals have higher GV compared with normal weight, nondiabetic individuals.
Selvin et al., 2021 [38]	pilot study of a prospective epidemiology study (ARIC); US	27 adults (8 with T2DM, 19 without diabetes)	October–November 2019	In persons without diabetes, there was a wide range of CGM parameters: range of mean glucose, 83.7–124.5 mg/dL, SD 12.2–27.3 mg/dL, CV 14.0–26.7%, and TIR 71.1–99.5%. In persons with diabetes, the range of mean CGM glucose was 105.5–223.0 mg/dL, SD, 22.3–86.6 mg/dL, CV 18.2–38.8%, TIR 38.7–98.3%. There was a high prevalence of hypoglycemia (glucose < 70 or <54 mg/dL) in both persons with and without diabetes.
Sezer et al., 2021 [43]	cross-sectional study; Turkey	27 non-diabetic, normotensive healthy subjects age over 18 years, BMI < 25 kg/m^2^ and absence of chronic disease (obesity, DM, lipid abnormalities, hypertension, liver, kidney and cv disease)		In the correlation analysis between glycemic variability parameters and BPV parameters, SD of 24-h SBP was correlated with the SD of MBG (r = 0.52, *p* = 0.006), MAGE (r = 0.49, *p* = 0.009), and MODD (r = 0.46, *p* = 0.015). SD of daytime SBP was correlated with, MAGE (r = 0.42, *p* = 0.03) and MODD (r = 0.43, *p* = 0.02). There is a correlation between glycemic variability and BPV variables in normoglycemic and normotensive healthy individuals.
Smith et al., 2021 [28]	parallel randomized controlled trial; Sweden	16 adults with obesity, self-perceived sedentary lifestyle, a sedentary occupation or unemployment, an age between 18–60 years and BMI 30–45 kg/m^2^	1 February 2017–31 July 2019	Mean (±SD) fasting glucose levels [−0.34 (±0.37) mmol/L] and daily glucose variation [%CV; −2% (±2.2%)] were reduced in FABS, suggesting a modest benefit for glycemic control that was most robust at higher volumes of daily activity.
Solomon et al., 2020 [33]	randomized, counter-balanced, controlled trial; UK	48 participants, aged 18–65 years, BMI 18–30 kg/m^2^, generally healthy and physically active	October 2017–November 2018	Walking and bodyweight exercises immediately after the meal improved mean, CV, and AUC glucose (*p* ≤ 0.05 vs. control), while standing immediately after the meal only improved AUC glucose (*p* ≤ 0.05 vs. control) and nearly improved mean glucose (*p* = 0.06). Mean, CV, and AUC glucose were not affected by standing, walking, or bodyweight exercise conducted immediately before, or 30 min after the meal (all *p* > 0.05 vs. control).
Teshima et al., 2020 [41]	observational two group, cohort study; Japan	18 patients with enlargement of cardiac silhouette CTR > 50%, NYHA II-IV and impaired systolic/diastolic function (EF < 50% or E/e > 14)		The average level and peak value of interstitial glucose concentrations, and the duration of hyperglycemia (≥140 mg/ dL) were not significantly different between Heart failure (+) and Heart failure (−). The duration of hypoglycemia (<80 mg/dL) was significantly longer and the trough value was significantly lower in Heart failure (+) compared with Heart failure (−).
Tey et al., 2017 [21]	randomized, crossover trial; Singapore	10 males, healthy, aged 21–50 years, BMI 18.5–25 kg/m^2^		No significant differences were found in mean 24-h glucose, iAUC and total AUC for glucose, and 24-h glycemic variability between the four test beverages. Twenty-four-hour glucose profiles did not differ between beverages sweetened with non-nutritive (artificial vs. natural) and nutritive sweeteners.
Timmer et al., 2022 [23]	randomized, three way, crossover trial; Netherlands	24 participants, aged 18–65 years, BMI 18.5–30 kg/m^2^, no reported health problems, no state of (pre)diabetes	15–24 February 2021 8–17 March 2021	The highest glycemic excursions to a standardized carbohydrate-rich snack (198 kcal) were observed in the morning, while a more dampened but prolonged response was observed in the evening.
Trim et al., 2023 [30]	randomized, controlled trial; UK	20 healthy, young (20–45 years old) males; selected by medical and psychological screening		Following long-term bed rest, fasting plasma insulin concentration increased 40% (*p* = 0.004) and glucose disposal during the HIEC decreased 24% (*p* < 0.001). Interstitial daily glucose total area under the curve (tAUC) from pre-to post-bed rest increased on average by 6% (*p* = 0.041), despite a 20 and 25% reduction in total caloric and carbohydrate intake, respectively. The nocturnal period (00:00–06:00) showed the greatest change to glycemia with glucose tAUC for this period increasing by 9% (*p* = 0.005). CGMS measures of daily glycemic variability (SD, J-Index, M- value and MAG) were not changed during bed rest.
Zhang et al., 2023 [25]	randomized, crossover trial; China	20 young adults (11 males, 9 females) with obesity (BMI ≥ 25 kg/m^2^), aged 18–35 years, who were sedentary (self-reported sedentary time > 8 h/day) and insufficiently active (<150 min moderate-intensity and/or 75 min vigorous intensity PA per week over the past 3 months)		The 4-h PPG incremental area under the curve (iAUC) was 12.1% ± 30.9% and 21.5% ± 21.5% smaller after CONT (*p* = 0.022) and ACCU (*p* < 0.001), respectively, than after SIT. PPG concentrations were lower during CONT at 30–60 min and during ACCU at 30–105 min after breakfast than during SIT (all *p* < 0.05). The 4-h plasma insulin and C-peptide iAUC, and mean amplitude of glycemic excursions were lower after CONT and ACCU than after SIT (all *p* < 0.05).
Zhang et al., 2021 [34]	randomized, controlled crossover trial; China	20 male participants, overweight or obese, BMI ≥ 23 kg/m^2^, aged 18–35 years, self-reported daily sedentary time > 8 h, and insufficient physical activity (measured by the Chinese version of the IPAQ Short Form)		Compared with SIT, the 4-h incremental AUCs (iAUCs) for plasma PPG (−0.6 mmol·L^−1^·h; *p* = 0.047) and insulin (−28.7%, *p* < 0.001) were reduced in 20 iP only, and C-peptide concentrations were lower after iP (−14.9%, *p* = 0.001) and 20 iP (−28.7%, *p* < 0.001). PPG reductions due to iP and 20 iP occurred only in men with a BMI > 27.5 kg/m^2^ (iP, −11.2%; 20 iP, −14.7%; *p* = 0.047) and higher glucose iAUC values during SIT (iP, −25.5%; 20 iP, −25.7%; *p* < 0.001).

## 4. Discussion

The aim of this paper was to systematically review the scope of literature regarding the application of CGM in cardiovascular disease prevention in non-diabetic individuals. Overall, due to CGM’s real-time visualization of glucose fluctuation in response to nutrition, physical activity, and other physiological parameters outlined previously, GV detection theoretically allows timely lifestyle intervention, which may potentially reduce the risk of cardiovascular disease progression and development [46,47,48].

This review supports the well-investigated change in blood glucose levels after food intake. Most reviewed studies using CGM in non-diabetics support the association between glycemic response measured with CGM, the GI, and the time of day of the meal. One study indicated no differential effect of incorporating higher or lower GI versions of carbohydrate-rich foods on daylong interstitial fluid glucose patterns measured with CGM [20]. In this study, lower or higher GI foods within mixed meals (unlimited quantities), small differences between low and high GI groups (66—high vs. 51 low GI), and a lack of 24-h glucose patterns may have influenced results. Moreover, although non-nutritive and nutritive sweetened beverages differ in their GI, no differences in glucose response have been reported [21].

Lower GI diets are associated with reduced glycemic variability and lower postprandial glucose levels. The amount and type of carbohydrates consumed, especially at breakfast, seem to influence postprandial glucose levels. Consistent meal timing, particularly not skipping breakfast, seems to play a crucial role in maintaining stable glucose levels. Some studies report conflicting results regarding GI and glucose response. A growing body of research is currently trying to establish analytical frameworks to group individuals according to glycemic response patterns, called glucotypes [49,50,51]. High variability of individual responses to the same food intake makes postprandial glucose predictions and glucotype definition difficult. This highlights the complexity of dietary impacts on glycemic control. However, early identification of glucose variability types could potentially serve as a tool for risk stratification for type 2 diabetes and other metabolic diseases [50].

Additionally, physical activity (PA), as well as inactivity, is connected to glycemic response in CGM. Several forms of PA, such as HIIT, moderate-intensity continuous exercise, walking, and activity breaks from sitting, are effective in improving glycemic control. Strategic timing of PA around meals seems to have an additional impact on PPG. Physical activity soon after a meal or strategically timed before peak glucose concentrations can improve PPG levels. The findings highlight the significance of incorporating specific types and timing of exercise to enhance glycemic control, particularly in populations at risk for diabetes.

Multiple studies collectively highlight the significance of glycemic variability (GV) as an associated critical factor in metabolic health. Cardiovascular risk factors other than nutrition and PA and their relationship to glucose variability were confirmed by the reviewed studies. The relationship between glycemic variability and cardiovascular parameters like HRV and BPV suggests a complex interplay between glucose regulation and cardiovascular health. CGM emerges as a valuable tool for assessing GV and its implications across different populations. However, all studies evaluated associations and further investigation regarding outcomes is warranted. Only one study dealt with outcome measures [45]. This study reported elevated exercise motivation with CGM-incorporated PA intervention.

The totality of evidence shows that CGM provides real-time tracking of glucose levels and is recommended as a tool for the early detection of abnormal glucose patterns. It offers a potential solution for normalizing glucose levels by encouraging behavioral changes, such as personalized diet adjustments and increased motivation for physical activity [48]. Especially regarding obesity, one of the greatest risk factors for the development of diabetes [52], CGM data provides biofeedback to support behavioral and lifestyle changes. Healthy behavior, as with increased PA and sleep, as well as portion control, could be helpful to delay or even prevent diabetes. Several biotech companies are marketing CGM to healthy individuals, warning of glucose spikes and promising weight management and PA integration aid [53,54,55]. Scientific proof, however, is lacking and emphasizes the necessity for outcome studies to justify an advantage for CV risk factor modulation by CGM. So far, no drawbacks for personal health have been reported. Infrequent tape allergy and skin trauma are rare and minor factors [51]. Stress or distress regarding constantly available data might negatively influence healthy eating and exercise behavior, triggering eating disorders or over-exaggerated exercise interventions. Also, glucose values highly vary between individuals. This makes interpretation of values for individuals with limited knowledge about blood glucose and the absence of norm values extremely difficult, resulting in possible undesirable effects. Furthermore, CGM measurements are delayed compared to blood glucose measurements due to the diffusion time of glucose to the subcutaneous interstitial fluid, which is significant in terms of rapidly changing glucose conditions such as PA, and therefore should also be taken into consideration in the reading/interpretation of CGM data.

## 5. Conclusions

Continuous glucose monitoring may offer significant potential benefits for cardiovascular prevention in healthy individuals without diabetes. By providing continuous data on glucose levels, CGM enables early identification of metabolic abnormalities, such as glycemic variability and postprandial hyperglycemia, known to be independent cardiovascular risk factors, and hence might support personalized and favorable lifestyle interventions in nutritional habits and physical activity.

Further research in long-term and outcome-oriented studies on glucose regulation in healthy, non-diabetic individuals and the influence of behavioral factors, is essential to fully understand and utilize its long-term impact on cardiovascular health.

## Data Availability

No new data were created or analyzed in this study.

## Supplementary Materials

The following supporting information can be downloaded at: https://www.mdpi.com/article/10.3390/s25010187/s1, PRISMA 2020 Checklist. Ref. [56] is cited in the Supplementary Materials.

## Abbreviations

The following abbreviations are used in this manuscript:

AUC	Area Under The Curve
iAUC	Incremental Area Under The Curve
BMI	Body Mass Index
BPV	Blood Pressure Variability
BWE	Bodyweight Exercise
CAD	Coronary Artery Disease
CGM	Continuous Glucose Monitoring
CMI	Continuous-Moderate Intensity
CV	Coefficient Of Variability
CVD	Cardiovascular Disease
EU	European Union
FABS	Frequent Activity Breaks From Sitting
GI	Glycemic Index
GV	Glycemic Variability
HbA1c	Hemoglobin A1c
HIIE	Home-Based High-Intensity Interval Exercise
HIIT	High-Intensity Interval Training
HRV	Heart Rate Variability
ICU	Intensive Care Unit
IGR	Impaired Glucose Regulation
IGT	Impaired Glucose Tolerance
IL-6	Interleukin-6
IMT	Intima-Media Thickness
ISF	Interstitial Fluid
MICE	Moderate-Intensity Continuous Exercise
NGT	Normal Glucose Tolerance
OSA	Obstructive Sleep Apnea
PA	Physical Activity
PPG	Postprandial Glucose
PPGP	Postprandial Glucose Peak Time
PRISMA	Preferred Reporting Items for Systematic Reviews and Meta-Analyses
T2DM	Type 2 Diabetes Mellitus
